# Is damage control surgery useful in the treatment of colorectal perforation? A single-center case–control study

**DOI:** 10.1097/MS9.0000000000000334

**Published:** 2023-03-25

**Authors:** Kosei Kunitatsu, Kentaro Ueda, Toru Nasu, Shuji Kawashima, Yuko Okishio, Seiya Kato

**Affiliations:** Department of Emergency and Critical Care Medicine, Wakayama Medical University, School of Medicine, Wakayama, Japan

**Keywords:** colorectal perforation, damage control surgery, emergency laparotomy, open abdominal management, peritonitis

## Abstract

Colorectal perforation with systemic peritonitis requires prompt surgical attention, and damage control surgery (DCS) is performed in patients with severe injuries. This study aimed to retrospectively investigate the efficacy of DCS in patients with colonic perforation.

**Materials and Methods:**

From January 2013 to December 2019, 131 patients with colorectal perforation underwent emergency surgery at our hospital. Among these, 95 patients required postoperative intensive care unit management and were included in this study; of these patients, 29 (31%) underwent DCS, and 66 (69%) underwent primary abdominal closure (PC).

**Results:**

Patients who underwent DCS had significantly higher Acute Physiology and Chronic Health Evaluation II (23.9 [19.5–29.5] vs. 17.6 [13.7–22]; *P*<0.0001) and Sequential Organ Failure Assessment (SOFA) (9 [7–11] vs. 6 [3–8]; *P*<0.0001) scores than did those who underwent PC. The initial operation time was significantly shorter for DCS than for PC (99 [68–112] vs. 146 [118–171]; *P*<0.0001). The 30-day mortality and colostomy rates were not significantly different between the two groups.

**Conclusions:**

The results suggest that DCS is useful in the management of acute generalized peritonitis caused by colorectal perforation.

## Introduction

Highlights

- Colorectal perforation with systemic peritonitis needs immediate surgical intervention.
- We examined the efficacy of damage control surgery (DCS) for colonic perforation.
- DCS controlled the infection source.
- DCS can manage colorectal perforation with systemic peritonitis.

Damage control surgery (DCS) has been discussed since World War II and is performed for severe trauma^1^. DCS with open abdominal management (OAM) is a management strategy used in severely injured patients, often with deranged physiology due to exsanguinating hemorrhage and excessive contamination^2^–^4^. Recently, a consensus paper was published by the World Society of Emergency Surgery on appropriate indications and technical recommendations for hemodynamic instability, excessive contamination, abdominal compartment syndrome, and mesenteric ischemia requiring reassessment of bowel viability in nontrauma patients^5^. In addition, some recent reports suggest that DCS for perforated diverticulitis can be performed safely and reduces the rate of colostomy^6^,^7^. However, some reports indicate that DCS has a significant potential for adverse outcomes and overuse of medical resources^8^,^9^. Although DCS is becoming increasingly popular, the indication for DCS in cases of colorectal perforation has not been established. We hypothesized that DCS might be an effective treatment modality in the surgical management of patients with septic shock associated with colorectal perforation. This retrospective study was conducted to evaluate the efficacy of DCS in nontrauma patients, particularly in cases of colonic perforation with generalized peritonitis.

## Materials and methods

### Ethical statement

The research protocol was approved by the Research Ethics Committee of Wakayama Medical University (approval number: 3124). The study was registered with the Research Registry (reserchregistry8662) in accordance with the Declaration of Helsinki and conducted in agreement with the guidelines of strengthening the reporting of cohort, cross-sectional, and case–control studies in surgery (STROCSS) 2021^10^.

### Patient selection

This was a single-center, retrospective study of patients who underwent emergency laparotomy for colorectal perforation in the Department of Emergency and Critical Care Medicine at our university hospital. All adult patients with colorectal perforation aged 20 years or older who underwent emergency laparotomy and were admitted to the ICU postoperatively between January 2013 and December 2019 were included. The exclusion criterion was colonic perforation not requiring postoperative ICU management. We divided patients into two groups: patients who required DCS (DCS group) and those who underwent primary abdominal closure (PC group). DCS was performed at the discretion of the surgeon, and the main indications were shock, severe contamination, abdominal compartment syndrome, and disseminated intravascular coagulation.

### Data collection

Demographic and clinical variables for all patients were manually collected from electronic medical and surgical records. The collected variables included age, sex, Sequential Organ Failure Assessment (SOFA) score, Acute Physiology and Chronic Health Evaluation II (APACHE II) score, initial operation time, perforation area, cause of perforation, and indication for DCS. The primary outcome was the 30-day survival rate. Secondary outcomes included the length of stay in the ICU, length of stay in the hospital, surgical site infection (SSI), unplanned relaparotomy, and the number of colostomies.

### Statistical analyses

Continuous data are reported as medians and interquartile ranges (IQRs), while categorical data are reported as frequencies and percentages. Statistical significance of the difference between DCS and PC groups was determined using Fisher’s exact test for categorical variables and the Wilcoxon rank-sum test for continuous variables. The JMP 16 software program (SAS Institute Inc., Cary, North Carolina, USA) was used for statistical analysis, and *P* less than 0.05 was considered to indicate statistical significance.

## Results

**Figure 1:**
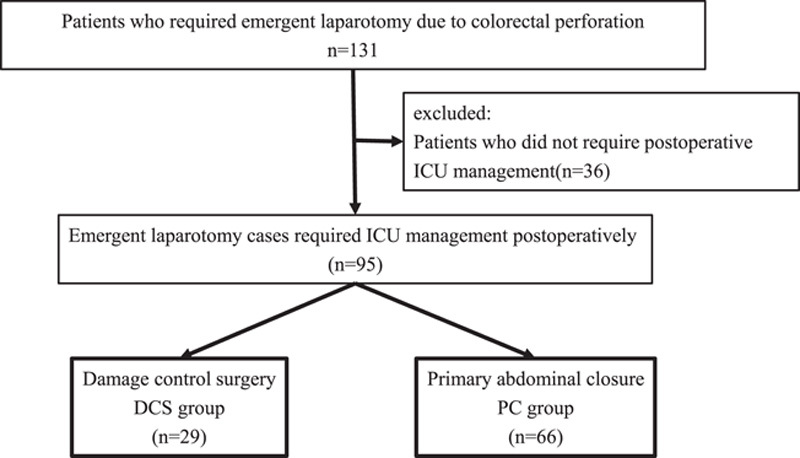
The study population selection flowchart. DCS, damage control surgery; PC, primary abdominal closure.

A total of 131 patients who required emergency laparotomy for colorectal perforation were enrolled in the study. Of these, 36 patients who did not require postoperative ICU management were excluded; the remaining 95 patients were finally included, with 29 (31%) in the DCS group and 66 (69%) in the PC group. The study population selection flowchart is shown in Figure 1.

The characteristics of patients undergoing emergency laparotomy for colorectal perforation are provided in Table 1. Both DCS and PC groups included mainly older adults (79 [71–86] vs. 75 [68–85] years; *P*=0.26), without a significant difference between the groups. The DCS group had a significantly higher APACHE II score (23.9 [19.5–29.5] vs. 17.6 [13.7–22]; *P*<0.0001) and a higher SOFA score (9 [7–11] vs. 6 [3–8]; *P*<0.0001) than did the PC group. The initial operation time was significantly shorter in the DCS group than in the PC group (99 [68–112] vs. 146 [118–171]; *P*<0.0001). There were no significant differences in the perforation area and cause of perforation between the DCS and PC groups. In both groups, the perforation site was in the sigmoid colon in about half of the patients, with the left colon from the descending colon to the rectum as the perforation site accounting for nearly 80% of cases. In the DCS group, the indication was determined by the surgeon for conditions such as shock vital signs (79%), excessive contamination (72%), mesenteric ischemia requiring reevaluation (24%), intra-abdominal hypertension (10%), and disseminated intravascular coagulation (17%). Twenty-seven of the 29 patients in the DCS group underwent temporary abdominal closure (TAC) with Barker’s vacuum packing technique^11^. In the remaining two patients, TAC was performed using the AbThera therapy system (KCI, now part of 3M Company, San Antonio, Texas, USA).

**Table 1:** Characteristics of patients undergoing emergency laparotomy for colorectal perforation

	DCS group (*n*=29)	PC group (*n*=66)	*P*
Age, years, median (IQR)	79 (71–86)	75 (68–85)	0.26
Male, *n* (%)	11 (38)	31 (47)	0.50
SOFA, median (IQR)	9 (7–11)	6 (3–8)	<0.0001
APACHE II score, median (IQR)	23.9 (19.5–29.5)	17.6 (13.7–22)	<0.0001
Initial operation time, median (IQR)	99 (68–112)	146 (118–171)	<0.0001
Perforation area			0.21
Rectum, *n* (%)	7 (24)	9 (14)	
Sigmoid, *n* (%)	14 (48)	34 (48)	
Descending, *n* (%)	1 (3)	8 (12)	
Transverse, *n* (%)	2 (7)	7 (11)	
Ascending and Cecum, *n* (%)	5 (17)	8 (12)	
Cause of perforation			0.93
Diverticulum, *n* (%)	8 (28)	23 (35)	
Cancer, *n* (%)	6 (21)	17 (26)	
Coprostasis, *n* (%)	7 (24)	9 (14)	
Necrosis, *n* (%)	2 (7)	4 (5)	
Others, *n* (%)	6 (21)	14 (21)	
Indication for DCS (overlapping)			
Shock vital, *n* (%)	23 (79)	NA	NA
Excessive contamination, *n* (%)	21 (72)	NA	NA
Mesenteric ischemia requiring revaluation, *n* (%)	7 (24)	NA	NA
Intra-abdominal hypertension, *n* (%)	3 (10)	NA	NA
Disseminated intravascular coagulation, *n* (%)	5 (17)	NA	NA

APACHE II , acute physiology and chronic health evaluation II; DCS, damage control surgery; IQR, interquartile range; PC, primary abdominal closure; SOFA sequential organ failure assessment.

Table 2 shows the outcome differences between the DCS and PC groups. There was no significant difference in the 30-day mortality rate between DCS and PC groups (10 vs. 6%; *P*=0.43). The length of stay in the ICU was significantly longer in the DCS group than in the PC group (8 [3.5–10] vs. 4 [2–5] days, *P*<0.0001); however, there was no significant difference in the length of stay in the hospital between the two groups (30 [14–42] vs. 26 [15–34] days; *P*=0.20). The DCS group had a lower frequency of unplanned relaparotomy (3 vs. 18%; *P*=0.10) than did the PC group; however, the difference failed to reach significance. Of the 29 patients in the DCS group, three died within 2 days after surgery and failed to achieve abdominal closure. The remaining 26 patients underwent fascial closure; the median number of operations required to close the abdomen was three (IQR, 2–4), and the time required to close the abdomen was 2 days (IQR, 1–5.25). Few patients required a long period of OAM and frequent laparotomy. Furthermore, in the DCS group, there was only one case of deep incisional SSI, requiring an unplanned relaparotomy (no other patients required an unplanned relaparotomy). In contrast, among the 66 patients in the PC group, there were three cases of stoma-related complications, two cases of bleeding, five cases of fascial dehiscence due to SSI, one case of anastomotic leakage, and one case of exploratory laparotomy. The DCS group had a higher rate of colostomy (92 vs. 79%; *P*=0.22) than did the PC group; however, the difference failed to reach significance.

**Table 2:** Outcome differences between the DCS and PC groups

	DCS group (*n*=29)	PC group (*n*=66)	*P*
30-day mortality, *n* (%)	3 (10)	4 (6)	0.43
Length of stay in ICU, median (IQR)	8 (3.5–10)	4 (2–5)	<0.0001
Length of stay in hospital, median (IQR)	30 (14–42)	26 (15–34)	0.17
SSI			
Superficial incisional SSI, *n* (%)	8 (28)	18 (27)	1
Deep incisional SSI, *n* (%)	1 (3)	5 (8)	0.66
Unplanned relaparotomy, *n* (%)	1 (3)	12 (18)	0.10
Colostomy^a^, *n* (%)	24 (92)	52 (79)	0.22

a Excluding three cases of death to failed primary fascial closure.

DCS, damage control surgery; IQR, interquartile range; PC, primary abdominal closure; SSI, surgical site infection.

## Discussion

Colorectal perforation with systemic peritonitis requires immediate surgical intervention. A high percentage of patients with this condition develop severe septic shock with multiple organ dysfunction^12^. The results of the present study support the effectiveness of DCS for patients with septic shock from colorectal perforation; patients who underwent DCS had a shorter initial operation time than patients who underwent PC, and the source of infection was controlled. Additionally, the number of laparotomies during the OAM period was relatively few in the DCS group. The results also suggest that DCS may reduce the number of unplanned relaparotomies.

In a summary of the evidence on OAM in trauma and nontrauma patients, Coccolini *et al*.^5^ stated that OAM is a treatment option for patients presenting with septic shock and severe peritonitis in the presence of the following conditions: abbreviated laparotomy due to severe physiological derangement, deferred intestinal anastomosis, planned second-look laparotomy for intestinal ischemia, extensive visceral edema with a risk of abdominal compartment syndrome, or persistent peritonitis source (failure of source control). The number of nontrauma patients undergoing OAM has been increasing; accordingly, the number of studies evaluating and discussing the outcomes of OAM in nontrauma patients has increased. Although OAM of nontrauma patients has become more frequent, the mortality rate among patients with colorectal perforation remains high and the utility of DCS has been debated.

Smith *et al*.^13^ conducted a propensity score-matched case-cohort study of three groups that underwent DCS for penetrating trauma, blunt trauma, and intraperitoneal sepsis; the group with intraperitoneal sepsis requiring DCS had the lowest rate of primary fascial closure, the highest rate of intraperitoneal complications, the longest time to closure, and highest 90-day mortality rate, compared to those in the other two groups. Similarly, in the present study, the APACHE II and SOFA scores were significantly higher in the DCS group than in the PC group. However, there was a trend toward high rates of primary fascial closure and few intra-abdominal complications with DCS. This suggests that, in our institution, DCS is appropriately performed in critically ill patients with septic shock, which is a good indication.

Sohn *et al*.^6^ showed that fecal peritonitis was a good indication for DCS because the septic focus could be rapidly removed during the initial surgery for peritonitis caused by perforated diverticulitis with generalized peritonitis. In cases of intra-abdominal sepsis, the minimum amount of surgery necessary to control the source of infection should be performed within a short time. It is important to perform TAC and promptly transfer the patient to intensive care management. In the present study, the initial operation time was shorter in the DCS group than in the PC group, suggesting that the septic focus was removed within a short time.

In DCS with OAM, patients with fascial closure difficulty may require component separation, planned ventral hernia, or mesh closure. However, fascial closure is the ideal technique for performing abdominal closure^5^. Poillucci *et al*.^14^ stated that negative pressure wound therapy was the best TAC method for reducing mortality and colostomy rates in patients undergoing OAM for severe intra-abdominal infection. In the present study, we were able to perform fascial closure in 26 of 29 patients (excluding three patients who died) who underwent OAM. The cause of death in the three patients was septic shock, and the patients were aged 90, 90, and 82 years. Quyn *et al*.^15^ reported that a high percentage of patients with peritonitis who underwent DCS developed enterocutaneous fistulas due to delayed fascial closure. Kao *et al*.^9^ compared OAM to primary closure after emergency laparotomy for peritonitis using propensity score matching and suggested that TAC was associated with a higher postoperative complication rate, in-hospital mortality rate, length of hospital stay, and costs. Therefore, OAM overuse has been debated. However, Hansraj *et al*.^16^ reported that 30% of patients who underwent second-look surgery required bowel resection and noted that OAM was a reasonable treatment strategy in selected patients. In our study, the one DCS case with OAM that required an unplanned relaparotomy was an abdominal wall dehiscence with a deep incisional SSI, which required intraperitoneal lavage and re-suturing. The patient has since had no recurrence. No cases of readmission for surgery-related complications were observed in patients who underwent DCS after surviving discharge or transfer. No cases of intestinal fistulas were observed, and the number of relaparotomies during the OAM period was relatively few, with a low unplanned relaparotomy rate. This suggests that DCS is effective for colorectal perforation with septic shock.

Tartaglia *et al*.^17^ reported that DCS for peritonitis due to diverticular perforation could reduce the rate of colostomy. Additionally, Cirocchi *et al*.^18^ reviewed and summarized the evidence on the benefits of DCS in perforated colonic diverticulitis; the authors found a weighted value of approximately 62.1% for achieving primary anastomosis with DCS for generalized peritonitis, with a primary leak rate of 4.7% and an all-cause mortality rate of 9.2%. In the subgroup analysis of diverticular perforation in this study, diverticular perforation occurred in eight patients in the DCS group and 23 in the PC group. The ages were 79 (69–82) years in the DCS group and 76 (66–84) years in the PC group, without any significant difference. Moreover, there were no significant differences in 30-day mortality, SSI, unscheduled reoperation, or colostomy rates. Only one death was observed in the PC group, which resulted from postoperative multiple organ failure. The patient was 89 years old. The present study had a high incidence of colostomy, which might be related to the higher age of the patients included in the present study compared to that in other studies. We believe that anastomosis should be performed carefully because anastomotic leakage is a serious complication in older adult patients and is associated with a high mortality rate. Bruns *et al*.^19^ found that the 6-month mortality rate for older nontrauma patients who underwent OAM was significantly higher than that for younger patients; the mortality rate was 28% for those aged less than 61 years, 38% for those aged 61–79 years, and 64% for those aged at least 79 years. A previous retrospective study^20^ also reported that, in older adult patients, DCS was associated with a higher risk of mortality. Nevertheless, in the present study, there were no significant differences in the length of hospital stay or SSI rate between the two groups, and a trend toward a lower rate of unplanned relaparotomy with DCS was observed. The results suggest that DCS is an effective strategy for treating severely ill patients with severe physiologic derangement and is not required for treating patients who are not severely ill.

Several limitations associated with the present study warrant mention. First, the analysis was conducted using retrospective data from a single center. Second, the study had a relatively low number of enrolled patients, and a selection bias could not be avoided. Third, this study was based on retrospective data; therefore, we could not examine medical costs. A large-scale multicenter study is required for a more thorough analysis.

## Conclusions

Although patients in the DCS group were more severely ill than those who underwent primary surgery, there was no difference in incisional SSI and 30-day mortality rates, and fascial closure was achieved in all patients, with a trend toward fewer reoperations. The initial operation time was also significantly reduced. Thus, DCS appears to be useful for treating severe acute generalized peritonitis caused by colorectal perforation.

## Ethical approval

The research protocol was approved by the Research Ethics Committee of Wakayama Medical University (approval number: 3124), and the study followed the tenets of the Declaration of Helsinki.

## Patient consent

Since this was a retrospective study, the requirement for informed consent from the study participants was waived.

## Sources of funding

No funding was received for this study.

## Author contribution

K.K.: principal investigator and writing the paper; K.U.: conceptualization, methodology, and supervision; T.N., S.K., Y.O., and S.K.: investigation.

## Conflicts of interest disclosure

The authors declare no conflicts of interest for this article.

## Provenance and peer review

Not commissioned and externally peer-reviewed.
